# Modifying functional brain networks in focal epilepsy by manual visceral-osteopathic stimulation of the vagus nerve at the abdomen

**DOI:** 10.3389/fnetp.2023.1205476

**Published:** 2023-07-13

**Authors:** Hendrik Lehnertz, Timo Broehl, Thorsten Rings, Randi von Wrede, Klaus Lehnertz

**Affiliations:** ^1^ BMT Internationale Akademie für Biodynamische Manuelle Therapie GmbH, Bühler, Switzerland; ^2^ Department of Epileptology, University of Bonn Medical Centre, Bonn, Germany; ^3^ Helmholtz Institute for Radiation and Nuclear Physics, University of Bonn, Bonn, Germany; ^4^ Interdisciplinary Center for Complex Systems, University of Bonn, Bonn, Germany

**Keywords:** electroencephalogram, epilepsy, functional brain network, vagus nerve, enteric nervous system, gut-brain axis

## Abstract

Non-invasive transcutaneous vagus nerve stimulation elicits similar therapeutic effects as invasive vagus nerve stimulation, offering a potential treatment alternative for a wide range of diseases, including epilepsy. Here, we present a novel, non-invasive stimulation of the vagus nerve, which is performed manually viscero-osteopathically on the abdomen (voVNS). We explore the impact of short-term voVNS on various local and global characteristics of EEG-derived, large-scale evolving functional brain networks from a group of 20 subjects with and without epilepsy. We observe differential voVNS-mediated alterations of these characteristics that can be interpreted as a reconfiguration and modification of networks and their stability and robustness properties. Clearly, future studies are necessary to assess the impact of such a non-pharmaceutical intervention on clinical decision-making in the treatment of epilepsy. However, our findings may add to the current discussion on the importance of the gut-brain axis in health and disease.

**Clinical Trial Registration**: https://drks.de/search/en/trial/DRKS00029914, identifier DRKS00029914

## 1 Introduction

Epilepsy is the third most common neurological disorder with a high impact on everyday life (World Health Organization, 2019). Not only the recurrent epileptic seizures themselves but also therapy-associated constraints as side effects and socio-legal consequences impair those affected. Though antiseizure medications (ASM), the first line treatment, provides seizure freedom in about two-thirds of people with epilepsy (PWE), for the other third several attempts at treatment are necessary and the search for alternatives is mandatory. Brain stimulation in epilepsy is an evolving field, with vagus nerve stimulation (VNS) being an established method (Groves and Brown, 2005; Beekwilder and Beems, 2010; Panebianco et al., 2022).

The vagus nerve is the longest nerve of the parasympathetic nervous system. It reaches from the vagal nuclei in the medulla to the colon, and since it strays in the entire abdominal cavity, it plays a key role in the communication between the brain and peripheral organs that are involved in the sensory detection and the autonomic control of visceral activity. Invasive vagus nerve stimulation (iVNS, with leads wrapped around the left vagus nerve at the carotid sheath) has been extensively studied since the 1990s and effectiveness as well as safety is well documented (Ben-Menachem, 2002). Recent studies with non-invasive transcutaneous auricular vagus nerve stimulation (taVNS, stimulating a cutaneous branch of the vagus nerve) support the idea of antiseizure effects with low risk of complications (von Wrede and Surges, 2021).

In addition to the aforementioned invasive and noninvasive approaches, there are other ways to stimulate the vagus nerve (Bailey and Bremer, 1938; Lanska, 2002; Cerritelli et al., 2016; Yuan and Silberstein, 2016a; Yuan and Silberstein, 2016b; Yuen and Sander, 2017; Payne et al., 2019; Capilupi et al., 2020; Okonogi and Sasaki, 2021; Goggins et al., 2022; Hilz, 2022). An alternative and so far not (or only insufficiently) investigated stimulation of the vagus nerve could be performed manually viscero-osteopathically on the abdomen (voVNS). Because of the high (approximately 75%) afferent fiber content of the vagus nerve, viscero-sensory information from the abdomen and thorax can be expected to exert more influence on the brain than *vice versa* (McMillin et al., 1999; Critchley and Harrison, 2013; Cerritelli et al., 2021). Also, the development of the primordial intestine, which precedes the development of the neural tube in time, underscores this directionality which supports the concept of a body-vagal-brain axis as part of the human physiolome (Lehnertz et al., 2020; Ivanov, 2021).

A recent study using so-called resting-state functional magnetic resonance imaging showed that osteopathic manual therapy is associated with changes in brain connectivity in healthy controls (Tramontano et al., 2020). However, it is unclear whether there are also voVNS-related immediate and specific changes in local and global properties of evolving functional brain networks (Lehnertz et al., 2014) derived from the ongoing electroencephalographic (EEG) activity in subjects with and without epilepsy. Observing such changes would contribute to improve understanding of vagus nerve stimulation in the context of non-pharmacological epilepsy therapy.

## 2 Materials and methods

### 2.1 Subjects

We screened subjects who were admitted to the ward of the Department of Epileptology at the University Hospital Bonn from June 2022 to February 2023 for suitability for this study. Inclusion criteria were clinical necessity (differential diagnosis or electrophysiological follow-up) for long-term video-EEG-recording and age 18 years and older. Exclusion criteria were actual or previous neurostimulation such as invasive or non-invasive vagus nerve stimulation or deep brain stimulation, progressive disease, seizures occurring within 24 h before the start of the study, insufficient German language capability, mental disability and incompetence to follow instructions. Demographic data were derived from subject reports and were completed before the study. Subjects were assigned to two different groups: epilepsy group (G1) and non-epilepsy group (G2). After being provided with written information and being given the opportunity to ask further questions, 25 subjects volunteered to participate and signed informed consent. All subjects were under stable CNS medication (if taking any) at least 24 h before stimulation, and no activation methods (such as hyperventilation or sleep deprivation) were applied at least 24 h before stimulation as well.

The study protocol had been approved by the ethics committee of the University of Bonn before the study has started. The study was included in the German clinical trial register (DRKS00029914), and all experiments were performed in accordance with relevant guidelines and regulations.

### 2.2 Manual visceral-osteopathic stimulation of the vagus nerve and electrophysiological recordings

In order to minimize the potential confounding influence of various ultradian rhythms on characteristics of functional brain networks (Lehnertz et al., 2021; von Wrede et al., 2022a) and on cardiac activity (Healy et al., 2021; Borovkova et al., 2022; Geng et al., 2022), we applied voVNS for 10 min in the early afternoon while subjects underwent a continuous 130-min electrophysiological (EEG and ECG) recording. The stimulation phase (“S”; manual visceral-osteopathic stimulation of the vagus nerve) was preceded by a 1-h pre-stimulation phase (baseline phase “B1”) and followed by a 1-h post-stimulation phase (baseline phase “B2”). During these phases subjects were at rest and awake.

In order to track possible changes of autonomic (heart, lung, skin, and guts) as well as features of the central nervous system [vigilance, mood/behavior, cognition and CNS-associated physiological symptoms (drowsiness, wakefulness, dizziness, double vision, balance)], a structured interview preceded and followed the study. In addition, the abdominal girth was measured at the level of the navel before the beginning of the study.

Stimulation was carried out with the same osteopathic hand position in all subjects. Hands were positioned on the abdomen to cover as much of the small intestine and colon as possible up to the left colon flexure (Cannon-Böhm point) in a way that achieves approximate anatomical accuracy. Using fascial release (Tozzi, 2012), a large portion of the small and large intestines (ascending and descending colon) was targeted. Then, the fascial dynamics were perceived, supported, and regulated to allow the organism to self-regulate the tension of the fascia of the aforementioned organs. The resulting improvement of their motility and peristalsis is thought to alter the vagus nerve transmission to the brain.

We recorded electroencephalograms (EEG) from 25 electrodes (Seeck et al., 2017) (Cz served as physical reference) and an electrocardiogram (ECG) from a modified lead-I configuration (two electrodes; placed at right upper and left lower chest). EEG and ECG data were sampled simultaneously at 256 Hz using a 16 bit analog-to-digital converter and were band-pass filtered offline (4th order Butterworth characteristic; EEG bandwidth: 1–45 Hz; ECG bandwidth: 3–25 Hz). To suppress contributions at the line frequency (50 Hz) a notch filter (3rd order) was applied. All recordings were visually inspected for strong artifacts (subject movements, amplifier saturation, or stimulation artifacts) and such data were excluded from further analyses.

### 2.3 Constructing and characterizing evolving functional brain networks

We constructed evolving, fully connected and weighted functional brain networks from a time-resolved synchronization analysis of the above mentioned EEG-recording, assessed important global and local characteristics of the networks, and tracked their changes over time. To enable comparability with our previous studies on VNS-induced alterations of functional brain networks (Rings et al., 2021; von Wrede et al., 2021; von Wrede et al., 2022a; von Wrede et al., 2022b), we proceeded as follows: we associated network vertices with brain regions sampled by the standard electrodes of the 10–20-system (Klem et al., 1999) and network edges with time-varying estimates of the strength of interactions between the dynamics of pairs of those brain regions, regardless of their anatomical connections. We derived these estimates from a moving-window analysis [non-overlapping windows; window duration 20 s (5120 data points)] of the mean phase coherence between all pairs of sampled brain regions (Mormann et al., 2000; Osterhage et al., 2007; Kuhnert et al., 2010; Fruengel et al., 2020). For subsequent analyses, we excluded windows containing artifacts (on average 24% of windows from B1, 11% from S, and 28% from B2).

We next assessed local and global network properties that were shown to be sensitive for a characterization of taVNS-induced alterations of functional brain networks (Rings et al., 2021; von Wrede et al., 2021; von Wrede et al., 2022a; von Wrede et al., 2022b) (see these references for details of analyses). Briefly, on the local network scale, we made use of two different and opposing centrality concepts to assess the relative importance of vertices and edges, namely a path-based and an interaction-strength-based concept. With both these concepts, non-redundant information about the role such constituents play in the larger network can be attained (Bröhl and Lehnertz, 2019; Bröhl and Lehnertz, 2022). As path-based centrality index, we employed betweenness centrality $C^{\text{B}}$ . A high $C^{\text{B}}$ value indicates a vertex/edge as central if it connects different regions of the network as a bridge. As interaction-strength-based centrality index, we employed eigenvector centrality $C^{\text{E}}$ . A high $C^{\text{E}}$ value indicates a constituent as central if the vertices/edges connected to it are central as well, therefore it reflects the influence of the vertex/edge on the network as a whole.

On the global network scale, we assessed the average clustering coefficient *C*, average shortest path length *L*, assortativity *A*, and synchronizability *S*. The average clustering coefficient *C* characterizes the network’s functional segregation, with *C* being lower, the more segregated the network is. The average shortest path length *L* characterizes the network’s functional integration; the more integrated the network, the lower is *L*. Functional segregation (integration) indexes independent (dependent) information processes between brain regions (Tononi et al., 1994). Assortativity *A* characterizes the network’s robustness (Newman, 2018). It reflects the tendency of edges to connect vertices with similar or equal properties. If edges preferentially connect vertices with similar (dissimilar properties), such networks are called assortative (disassortative). Disassortative networks are more vulnerable to perturbations and appear to be easier to synchronize than assortative networks. Synchronizability *S* characterizes the network’s stability (Arenas et al., 2008). It assesses the network’s propensity (or vulnerability) to get synchronized by an admissible input activation: the higher *S*, the more easily can the synchronized state be perturbed.

### 2.4 Assessing possible voVNS-related alterations of heart rate variability

Fast fluctuations and slow trends were already reduced in the filtered ECG time series, and in a next step, we smoothed these time series (convolution with a Hamming kernel; kernel width: 10 datapoints) to facilitate automated identification of local maxima. A local maximum was accepted as a heart beat (peak of R-wave) if its amplitude value exceeded any other local maxima in a window of 400 ms duration centered around that maximum. We then calculated heart rate (HR) and heart rate variability (HRV) from beat-to-beat intervals as the inverse of the median resp. as the standard deviation of the intervals for successive, non-overlapping 5-min periods (Shaffer and Ginsberg, 2017). Eventually, we assigned these data to the three phases (B1, S, B2).

### 2.5 Statistics

For each phase of the examination schedule (B1, S, and B2), we investigated whether the two subject groups (G1 and G2) presented with different local and global network characteristics and with different heart rates, resp. heart rate variabilities (mean values of characteristics and rates from each phase; Mann-Whitney U-test; *p* < 0.05). For each subject, we investigated if the aforementioned brain network and cardiac characteristics differed between the phases of the examination schedule (distributions of characteristics and rates from each phase; Mann-Whitney U-test; *p* < 0.05). In order to distinguish cases that “responded” to the stimulation from “non-responding” cases we repeated the latter analysis on a single subject level. We considered a subject as a responder, if network characteristics in at least three 10-min windows during the baseline phase B1 differed from the ones during the stimulation phase S (Kolmogorov–Smirnov test; *p* < 0.05). All *p*-values were corrected for multiple comparisons using the Bonferroni method. We note that abdominal girth appeared to have no influence on whether or not a subject is a responder (Fisher’s exact test).

## 3 Results

From the twenty-five eligible subjects, three subjects had to be excluded from the study prior to stimulation due to medical reasons, another two subjects had to be excluded due to EEG data quality. Data from twenty subjects (4 females; age 19–59 years, median 36.5 years) qualified for subsequent analyses. Ten subjects suffered from focal epilepsy (G1: 3 females; age 22–56 years, median 37.0 years; duration of epilepsy 0–40 years, median 7.0 years): eight of them from structural focal epilepsy with different anatomical onset locations (semiology, EEG, MRI) and two from a focal epilepsy of unknown etiology. The other ten subjects did not suffer from epilepsy and had never experienced seizures before (G2: 1 female; age 19–59 years, median 36.5 years).

### 3.1 Impact of voVNS on local network characteristics

**FIGURE 1:**
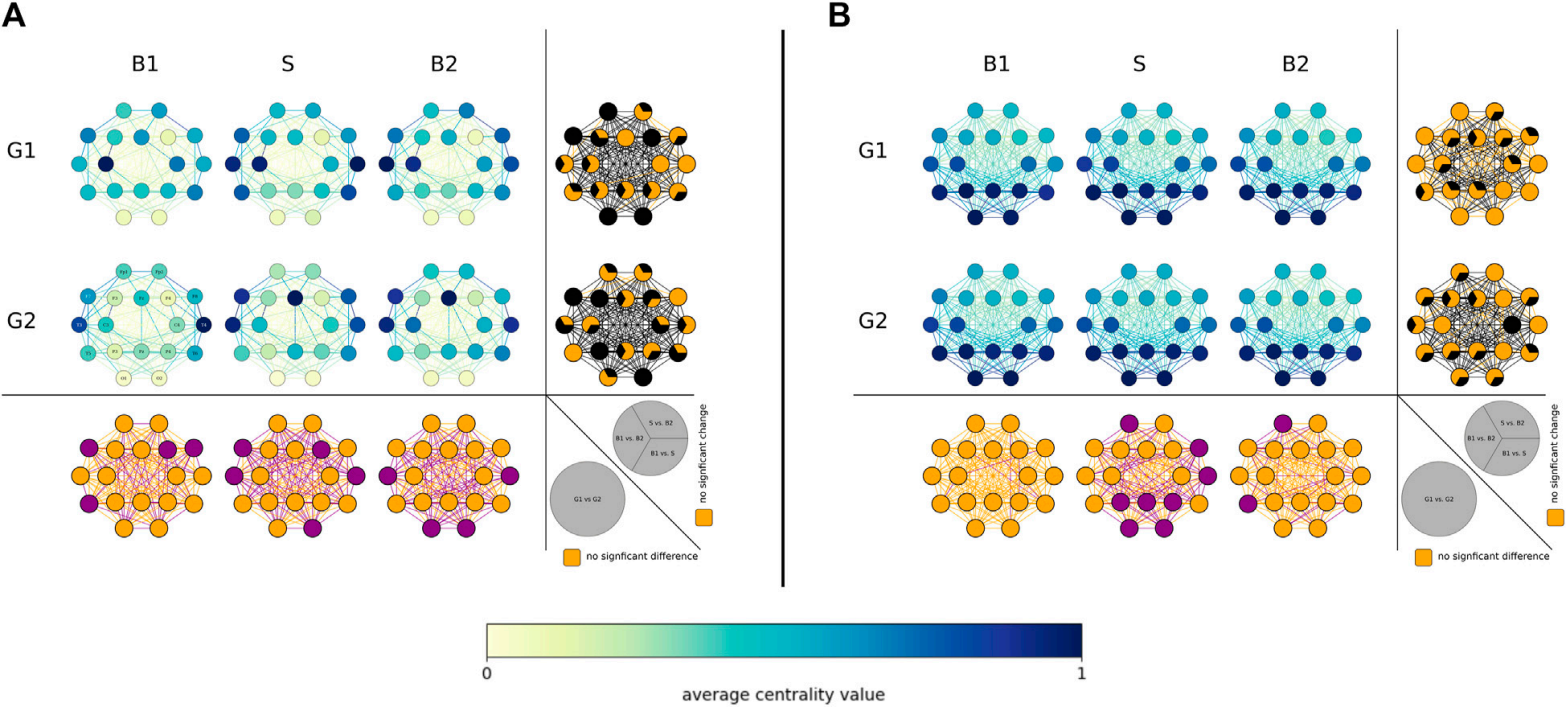
Distributions of voVNS-related alterations in local network characteristics in the epilepsy group (G1, top) and the non-epilepsy group (G2, middle). **(A)** Betweenness centrality $C^{\text{B}}$ and **(B)** eigenvector centrality $C^{\text{E}}$ in the three investigated phases (B1 = pre-stimulation baseline 1, S = stimulation, B2 = post-stimulation baseline 2). Network vertices arranged according the international 10–20 system for EEG-recording (electrode naming see leftmost plot for G2). Color coding of vertices and edges according to the average centrality values (temporal means and groups means). Bottom: Difference between groups (G1, G2) for local network characteristics in the three investigated phases. Orange: no significance, purple: significant difference (*p* < 0.05). Right side each plot: differences between phases (B1, S, B2) for local network characteristics in the investigated groups. Orange: no significance, black: significant change (*p* < 0.05).

Depending on the employed centrality concept we obtained different results on the population sample level (see Figure 1), which is in line with previous studies (Rings et al., 2021; von Wrede et al., 2022b).

In both groups, vertex betweenness centrality stressed vertices associated with fronto-centro-temporal brain regions (left side slightly accentuated) as most important (high $C^{\text{B}}$ values), while vertex eigenvector centrality highlighted vertices associated with posterior brain regions as most important (high $C^{\text{E}}$ values). Apart from some few, locally mostly unspecific differences, most important vertices differed significantly neither between groups nor between phases. As regards most important edges, none of the employed edge centrality concepts stressed a definite spatial pattern of differences, neither between groups nor between phases.

Nevertheless, we observed significant voVNS-mediated changes between groups (G1 and G2) as well as between phases (B1, S, and B2). Within each phase, the groups differed significantly in some few specific network constituents. Within each group, VoVNS exerted an immediate (from phase B1 to phase S and from phase S to phase B2) and an enduring (from phase B1 to phase B2) importance-modifying effect on some — rather few — specific constituents (see Figures 1A, B; rightmost columns) which, however, did not appear to be related to specific structural aspects. Overall and on the level of the population sample investigated here, voVNS thus appeared to have an only negligible immediate and enduring impact on the importance hierarchy as yielded by the local network characteristics.

### 3.2 Impact of voVNS on global network characteristics

On the population sample level, we observed for both groups comparable topological network characteristics (average clustering coefficient *C* and average shortest path length *L*) as well as comparable stability and robustness characteristics (synchronizability *S* and assortativity *A*) during all phases of the examination schedule. There were also no significant differences between global network characteristics from each phase in each subject group (data not shown).

**FIGURE 2:**
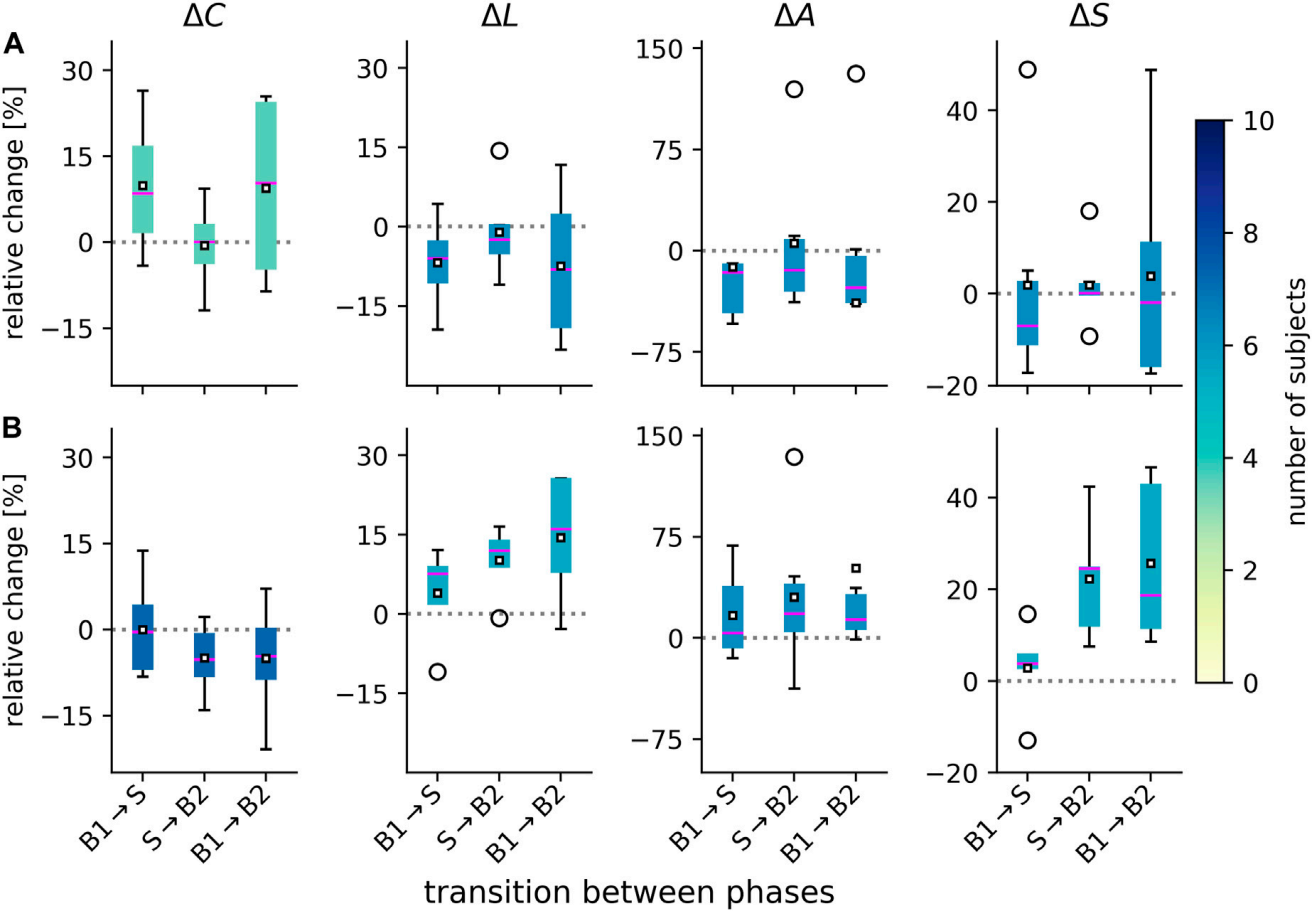
Distributions of voVNS-related alterations in global network characteristics in the epilepsy group [G1, **(A)**] and the non-epilepsy group [G2, **(B)**]. Boxplots of relative changes Δ in network characteristics (average clustering coefficient *C*, average shortest path length *L*, assortativity *A*, and synchronizability *S*). Relative changes calculated as Δ = (*M*_*l*_ − *M*_*k*_)/*M*_*k*_, where *M*_*k*_ and *M*_*l*_ denote placeholders for the temporal means of the respective characteristics from phase *k* and phase *l*. During phase 1 (B1), network characteristics attained the following values in G1: *C* = 0.31 ± 0.04, *L* = 3.60± 0.35, *A* = 0.21 ± 0.11, *S* = 3.22 ± 0.39, and in G2: *C* = 0.35 ± 0.04, *L* = 3.08 ± 0.37, *A* = 0.19 ± 0.05, *S* = 2.63 ± 0.20. There were no significant differences between groups for the three phases (B1, S, B2). Bottom and top of a box are the first and third quartiles, and the magenta band and the black square are the median and the mean of the distribution. Outliers are marked by a ◦ sign. Color coding of boxes according to the number of subjects for whom we obtained significant changes in global network characteristics on a per-subject base.

From prior studies on the impact of taVNS on evolving functional brain networks (Rings et al., 2021; von Wrede et al., 2021; von Wrede et al., 2022b), we suspected that not all subjects may exhibit voVNS-mediated changes of their networks. We therefore only focused on those subjects for whom we identified significant changes of their network characteristics (see Figure 2). The subject groups presented with a different pattern of change.

VoVNS exerted an immediate (from phase B1 to phase S) topology-modifying effect on the networks of the responders in the epilepsy group (G1), namely they became less segregated and more integrated [average clustering coefficient *C* increased (relative change of median values: 8.5%) while the average shortest path length *L* decreased (−5.9%)]. Changes were negligible when networks transit back to the post-stimulation baseline (from phase S to phase B2). The responders in the non-epilepsy group (G2) only presented with slightly increased average shortest path length *L* (7.7%), which would indicate a less integrated network upon stimulation. In contrast to G1, their networks became more segregated and even less integrated when transiting back to the post-stimulation baseline (*C* decreased by −5.2%; *L* increased by 12.0%), which possibly points to enduring effect of voVNS in this group. Indeed, a comparison of network characteristics from the phases prior to (B1) and after the stimulation (B2), allowed us to identify an enduring effect in the epilepsy group (G1) that rendered their network less segregated (*C* increased by 10.3%) and more integrated (*L* decreased by −8.1%). We observed opposing enduring effects in the non-epilepsy group (G2): their networks became more segregated (*C* decreased by −4.6%) and more integrated (*L* increased by 16.1%).

An opposing effect between groups was also seen for network robustness. VoVNS led to an immediate and enduring robustness-decreasing effect on the networks in the epilepsy group [assortativity *A* decreased between phases B1 and S (−16.2%) and between phases B1 and B2 (−27.5%)]. In the non-epilepsy group, an immediate and enduring robustness-increasing effect was observed [assortativity *A* increased between phases B1 and S (3.6%) and between phases B1 and B2 (13.5%)]. Regarding network stability, we observed voVNS in G1 to slightly decrease the networks’ vulnerability of the synchronized state to get perturbed (*S* decreased between B1 and S by −7.0% as well as between B1 and B2 by −1.9%). In G2, we observed a more pronounced, vulnerability-increasing effect (*S* increases between B1 and S by 3.9% as well as between B1 and B2 by 18.7%).

### 3.3 Impact of voVNS on heart rate variability

**FIGURE 3:**
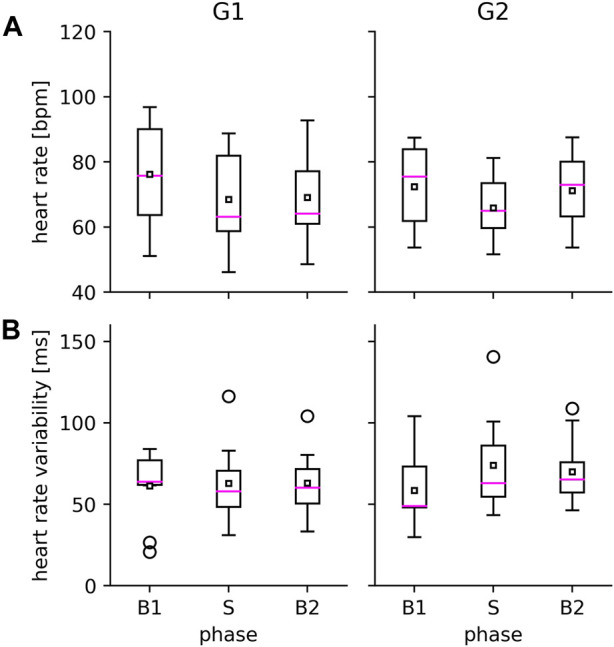
VoVNS-related alterations in heart rate **(A)** and heart rate variability **(B)** in the epilepsy group (G1, left) and the non-epilepsy group (G2, right). Properties of boxplot as in Figure 2.

There were no significant differences between cardiac characteristics of the two groups in the respective phases (data not shown). Although heart rate slightly decreased, on average, during voVNS (see Figure 3) in both groups, this was not significant. Also, there were no voVNS-related changes in heart rate variability.

### 3.4 Structured interview

None of the subjects voiced complains during voVNS. None of the subjects experienced subjective changes of the autonomic system (heart, lung, skin). One subject from G1 reported diarrhea and one subject from G2 experienced feelings of elevated peristaltic. One subject from G1 reported a discrete improvement of mood and four subjects from G2 reported to feel more awake after the stimulation. None of the epilepsy subjects reported changes of central-nervous-system-associated symptoms.

## 4 Discussion

We demonstrated–to our knowledge for the first time–that manual viscero-osteopathic stimulation of the vagus nerve on the abdomen (voVNS) induces measurable immediate changes in local and global properties of evolving functional brain networks in subjects with and without epilepsy. Our findings could thus add to the current discussion on the importance of the gut-brain axis (Bonaz et al., 2018; Rebollo et al., 2018; Chuyue et al., 2020; Mayer et al., 2022) not only for various physiological regulatory mechanisms but also for gastrointestinal, immunological, and neurological disorders, including epilepsy (Dahlin and Prast-Nielsen, 2019; Chatzikonstantinou et al., 2021; Ding et al., 2021; Iannone et al., 2022; Sinha et al., 2022).

In line with previous studies on the impact of transcutaneous auricular vagus nerve stimulation (taVNS) on such networks (Rings et al., 2021; von Wrede et al., 2021; von Wrede et al., 2022b), we observed that not all subjects presented with voVNS-mediated changes of their networks. For subjects that responded to the stimulation and on the local network scale, voVNS induced significant but unspecific modifications of vertex- and edge-related characteristics (edge and vertex centralities) throughout the network. This corroborates the popular view of VNS rather unspecifically and globally activating various brain structures (Vonck et al., 2001; Groves and Brown, 2005; Yap et al., 2020; Carron et al., 2022; Goggins et al., 2022). On the global network scale, we observed voVNS to differentially modify (both immediately and enduringly) topological as well as stability- and robustness-associated network properties in subjects with and without epilepsy. Similar findings were also reported for taVNS (von Wrede et al., 2022b). When comparing taVNS- and voVNS-mediated network modifications, more similarities could be observed for subjects without epilepsy than for subject with epilepsy, particularly with regard to enduring topological as well as stability- and robustness-associated network properties. Whether such similarities provide first clues for a possible mechanism of action of voVNS remains speculative and calls for further, sham-controlled studies on larger subject groups.

The viscero-osteopathic vagus nerve stimulation seemed not to disrupt the cardiac autonomic function, neither in subjects without nor in subjects with epilepsy. For the latter, similar observations were made for chronic invasive stimulation of the left cervical vagus nerve as an add-on treatment for medically refractory epilepsy (Constantinescu et al., 2019; Wu et al., 2021). Studies on cardiac effects of short-term (minutes to hours) taVNS have so far been performed in healthy subjects only, however, with conflicting findings (De Couck et al., 2017; Badran et al., 2018; Capilupi et al., 2020; Keute et al., 2021; Machetanz et al., 2021a; Machetanz et al., 2021b; Wolf et al., 2021; Forte et al., 2022). These can probably be related to stimulation-parameter-dependent influences that act on brain-heart couplings (Cerritelli et al., 2021; Keute et al., 2021).

To summarize, our findings provide initial evidence for viscero-osteopathic vagus nerve stimulation as a possible alternative, non-invasive option for non-pharmacological epilepsy therapy.

## Data Availability

The datasets presented in this article are not readily available because they contain information that could compromise the privacy of research participants. Requests to access the datasets should be directed to klaus.lehnertz@ukbonn.de.

## References

[B1] Arenas A. Díaz-Guilera A. Kurths J. Moreno Y. Zhou C. (2008). Synchronization in complex networks. Phys. Rep. 469, 93–153. 10.1016/j.physrep.2008.09.002

[B2] Badran B. W. Mithoefer O. J. Summer C. E. LaBate N. T. Glusman C. E. Badran A. W. (2018). Short trains of transcutaneous auricular vagus nerve stimulation (taVNS) have parameter-specific effects on heart rate. Brain Stimul. 11, 699–708. 10.1016/j.brs.2018.04.004 29716843 PMC6536129

[B3] Bailey P. Bremer F. (1938). A sensory cortical representation of the vagus nerve: With a note on the effects of low blood pressure on the cortical electrogram. J. Neurophysiol. 1, 405–412. 10.1152/jn.1938.1.5.405

[B4] Beekwilder J. Beems T. (2010). Overview of the clinical applications of vagus nerve stimulation. J. Clin. Neurophysiol. 27, 130–138. 10.1097/WNP.0b013e3181d64d8a 20505378

[B5] Ben-Menachem E. (2002). Vagus-nerve stimulation for the treatment of epilepsy. Lancet Neurol. 1, 477–482. 10.1016/s1474-4422(02)00220-x 12849332

[B6] Bonaz B. Bazin T. Pellissier S. (2018). The vagus nerve at the interface of the microbiota-gut-brain axis. Front. Neurosci. 12, 49. 10.3389/fnins.2018.00049 29467611 PMC5808284

[B7] Borovkova E. I. Prokhorov M. D. Kiselev A. R. Hramkov A. N. Mironov S. A. Agaltsov M. V. (2022). Directional couplings between the respiration and parasympathetic control of the heart rate during sleep and wakefulness in healthy subjects at different ages. Front. Netw. Physiol. 2, 942700. 10.3389/fnetp.2022.942700 36926072 PMC10013057

[B8] Bröhl T. Lehnertz K. (2022). A straightforward edge centrality concept derived from generalizing degree and strength. Sci. Rep. 12, 4407. 10.1038/s41598-022-08254-5 35292696 PMC8922089

[B9] Bröhl T. Lehnertz K. (2019). Centrality-based identification of important edges in complex networks. Chaos 29, 033115. 10.1063/1.5081098 30927842

[B10] Capilupi M. J. Kerath S. M. Becker L. B. (2020). Vagus nerve stimulation and the cardiovascular system. Cold Spring Harb. Perspect. 10, a034173. 10.1101/cshperspect.a034173 PMC6996447 31109966

[B11] Carron R. Roncon P. Lagarde S. Dibué M. Zanello M. Bartolomei F. (2022). Latest views on the mechanisms of action of surgically implanted cervical vagal nerve stimulation in epilepsy. Neuromodulation 26, 498–506. 10.1016/j.neurom.2022.08.447 36064522

[B12] Cerritelli F. Chiacchiaretta P. Gambi F. Saggini R. Perrucci M. G. Ferretti A. (2021). Osteopathy modulates brain–heart interaction in chronic pain patients: An ASL study. Sci. Rep. 11, 4556. 10.1038/s41598-021-83893-8 33633195 PMC7907192

[B13] Cerritelli F. Ruffini N. Lacorte E. Vanacore N. (2016). Osteopathic manipulative treatment in neurological diseases: Systematic review of the literature. J. Neurol. Sci. 369, 333–341. 10.1016/j.jns.2016.08.062 27653920

[B14] Chatzikonstantinou S. Gioula G. Kimiskidis V. K. McKenna J. Mavroudis I. Kazis D. (2021). The gut microbiome in drug-resistant epilepsy. Epilepsia open 6, 28–37. 10.1002/epi4.12461 33681645 PMC7918308

[B15] Chuyue D. Y. Xu Q. J. Chang R. B. (2020). Vagal sensory neurons and gut-brain signaling. Curr. Opin. Neurobiol. 62, 133–140. 10.1016/j.conb.2020.03.006 32380360 PMC7560965

[B16] Constantinescu V. Matei D. Constantinescu I. Cuciureanu D. I. (2019). Heart rate variability and vagus nerve stimulation in epilepsy patients. Transl. Neurosci. 10, 223–232. 10.1515/tnsci-2019-0036 31497318 PMC6708288

[B17] Critchley H. D. Harrison N. A. (2013). Visceral influences on brain and behavior. Neuron 77, 624–638. 10.1016/j.neuron.2013.02.008 23439117

[B18] Dahlin M. Prast-Nielsen S. (2019). The gut microbiome and epilepsy. EBioMedicine 44, 741–746. 10.1016/j.ebiom.2019.05.024 31160269 PMC6604367

[B19] De Couck M. Cserjesi R. Caers R. Zijlstra W. Widjaja D. Wolf N. (2017). Effects of short and prolonged transcutaneous vagus nerve stimulation on heart rate variability in healthy subjects. Aut. Neurosci. 203, 88–96. 10.1016/j.autneu.2016.11.003 28017263

[B20] Ding M. Lang Y. Shu H. Shao J. Cui L. (2021). Microbiota–gut–brain axis and epilepsy: A review on mechanisms and potential therapeutics. Front. Immun. 12, 742449. 10.3389/fimmu.2021.742449 PMC8542678 34707612

[B21] Forte G. Favieri F. Leemhuis E. De Martino M. L. Giannini A. M. De Gennaro L. (2022). Ear your heart: Transcutaneous auricular vagus nerve stimulation on heart rate variability in healthy young participants. PeerJ 10, e14447. 10.7717/peerj.14447 36438582 PMC9686410

[B22] Fruengel R. Bröhl T. Rings T. Lehnertz K. (2020). Reconfiguration of human evolving large-scale epileptic brain networks prior to seizures: An evaluation with node centralities. Sci. Rep. 10, 21921. 10.1038/s41598-020-78899-7 33318564 PMC7736584

[B23] Geng D. Yang K. Fu Z. Zhang Y. Wang C. An H. (2022). Circadian stage-dependent and stimulation duration effects of transcutaneous auricular vagus nerve stimulation on heart rate variability. Plos one 17, e0277090. 10.1371/journal.pone.0277090 36327249 PMC9632923

[B24] Goggins E. Mitani S. Tanaka S. (2022). Clinical perspectives on vagus nerve stimulation: Present and future. Clin. Sci. 136, 695–709. 10.1042/CS20210507 PMC9093220 35536161

[B25] Groves D. A. Brown V. J. (2005). Vagal nerve stimulation: A review of its applications and potential mechanisms that mediate its clinical effects. Neurosci. Biobehav. Rev. 29, 493–500. 10.1016/j.neubiorev.2005.01.004 15820552

[B26] Healy K. L. Morris A. R. Liu A. C. (2021). Circadian synchrony: Sleep, nutrition, and physical activity. Front. Netw. Physiol. 1, 732243. 10.3389/fnetp.2021.732243 35156088 PMC8830366

[B27] Hilz M. J. (2022). Transcutaneous vagus nerve stimulation–A brief introduction and overview. Auton. Neurosci. 243, 103038. 10.1016/j.autneu.2022.103038 36201901

[B28] Iannone L. F. Gómez-Eguílaz M. De Caro C. (2022). Gut microbiota manipulation as an epilepsy treatment. Neurobiol. Dis. 174, 105897. 10.1016/j.nbd.2022.105897 36257595

[B29] Ivanov P. C. (2021). The new field of network physiology: Building the human physiolome. Front. Netw. Physiol. 1, 711778. 10.3389/fnetp.2021.711778 36925582 PMC10013018

[B30] Keute M. Machetanz K. Berelidze L. Guggenberger R. Gharabaghi A. (2021). Neuro-cardiac coupling predicts transcutaneous auricular vagus nerve stimulation effects. Brain Stimul. 14, 209–216. 10.1016/j.brs.2021.01.001 33422683

[B31] Klem G. Lüders H. Jasper H. Elger C. (1999). The ten-twenty electrode system of the international Federation. The international Federation of clinical Neurophysiology. Electroencephalogr. Clin. Neurophysiol. Suppl. 52, 3–6. 10590970

[B32] Kuhnert M.-T. Elger C. E. Lehnertz K. (2010). Long-term variability of global statistical properties of epileptic brain networks. Chaos 20, 043126. 10.1063/1.3504998 21198096

[B33] Lanska D. J. (2002). J.L. Corning and vagal nerve stimulation for seizures in the 1880s. Neurology 58, 452–459. 10.1212/wnl.58.3.452 11839848

[B34] Lehnertz K. Ansmann G. Bialonski S. Dickten H. Geier C. Porz S. (2014). Evolving networks in the human epileptic brain. Phys. D. 267, 7–15. 10.1016/j.physd.2013.06.009

[B35] Lehnertz K. Bröhl T. Rings T. (2020). The human organism as an integrated interaction network: Recent conceptual and methodological challenges. Front. Physiol. 11, 598694. 10.3389/fphys.2020.598694 33408639 PMC7779628

[B36] Lehnertz K. Rings T. Bröhl T. (2021). Time in brain: How biological rhythms impact on EEG signals and on EEG-derived brain networks. Front. Netw. Physiol. 1, 755016. 10.3389/fnetp.2021.755016 36925573 PMC10013076

[B37] Machetanz K. Berelidze L. Guggenberger R. Gharabaghi A. (2021a). Brain–heart interaction during transcutaneous auricular vagus nerve stimulation. Front. Neurosci. 15, 632697. 10.3389/fnins.2021.632697 33790736 PMC8005577

[B38] Machetanz K. Berelidze L. Guggenberger R. Gharabaghi A. (2021b). Transcutaneous auricular vagus nerve stimulation and heart rate variability: Analysis of parameters and targets. Aut. Neurosci. 236, 102894. 10.1016/j.autneu.2021.102894 34662844

[B39] Mayer E. A. Nance K. Chen S. (2022). The gut–brain axis. Annu. Rev. Med. 73, 439–453. 10.1146/annurev-med-042320-014032 34669431

[B40] McMillin D. L. Richards D. G. Mein E. A. Nelson C. D. (1999). The abdominal brain and enteric nervous system. J. Altern. Complement. Med. 5, 575–586. 10.1089/acm.1999.5.575 10630351

[B41] Mormann F. Lehnertz K. David P. Elger C. E. (2000). Mean phase coherence as a measure for phase synchronization and its application to the EEG of epilepsy patients. Phys. D. 144, 358–369. 10.1016/S0167-2789(00)00087-7

[B42] Newman M. (2018). Networks. Oxford University Press.

[B43] Okonogi T. Sasaki T. (2021). Optogenetic Manipulation of the vagus nerve. Singapore: Springer Singapore, 459–470. 10.1007/978-981-15-8763-4_30 33398833

[B44] Osterhage H. Mormann F. Staniek M. Lehnertz K. (2007). Measuring synchronization in the epileptic brain: A comparison of different approaches. Int. J. Bifurc. Chaos Appl. Sci. Eng. 17, 3539–3544. 10.1142/s0218127407019330

[B45] Panebianco M. Rigby A. Marson A. G. (2022). Vagus nerve stimulation for focal seizures. Cochrane Database Syst. Rev. 7, CD002896. 10.1002/14651858.CD002896.pub3 35833911 PMC9281624

[B46] Payne S. C. Furness J. B. Stebbing M. J. (2019). Bioelectric neuromodulation for gastrointestinal disorders: Effectiveness and mechanisms. Nat. Rev. Gastroenterol. Hepatol. 16, 89–105. 10.1038/s41575-018-0078-6 30390018

[B47] Rebollo I. Devauchelle A.-D. Béranger B. Tallon-Baudry C. (2018). Stomach-brain synchrony reveals a novel, delayed-connectivity resting-state network in humans. eLife 7, e33321. 10.7554/eLife.33321 29561263 PMC5935486

[B48] Rings T. von Wrede R. Bröhl T. Schach S. Helmstaedter C. Lehnertz K. (2021). Impact of transcutaneous auricular vagus nerve stimulation on large-scale functional brain networks: From local to global. Front. Physiol. 12, 700261. 10.3389/fphys.2021.700261 34489724 PMC8417898

[B49] Seeck M. Koessler L. Bast T. Leijten F. Michel C. Baumgartner C. (2017). The standardized EEG electrode array of the IFCN. Clin. Neurophysiol. 128, 2070–2077. 10.1016/j.clinph.2017.06.254 28778476

[B50] Shaffer F. Ginsberg J. P. (2017). An overview of heart rate variability metrics and norms. Front. Publ. Health 5, 258. 10.3389/fpubh.2017.00258 PMC5624990 29034226

[B51] Sinha N. Joshi R. B. Sandhu M. R. S. Netoff T. I. Zaveri H. P. Lehnertz K. (2022). Perspectives on understanding aberrant brain networks in epilepsy. Front. Netw. Physiol. 2, 868092. 10.3389/fnetp.2022.868092 36926081 PMC10013006

[B52] Tononi G. Sporns O. Edelman G. M. (1994). A measure for brain complexity: Relating functional segregation and integration in the nervous system. Proc. Natl. Acad. Sci. 91, 5033–5037. 10.1073/pnas.91.11.5033 8197179 PMC43925

[B53] Tozzi P. (2012). Selected fascial aspects of osteopathic practice. J. Bodyw. Mov. Ther. 16, 503–519. 10.1016/j.jbmt.2012.02.003 23036882

[B54] Tramontano M. Cerritelli F. Piras F. Spanò B. Tamburella F. Piras F. (2020). Brain connectivity changes after osteopathic manipulative treatment: A randomized manual placebo-controlled trial. Brain Sci. 10, 969. 10.3390/brainsci10120969 33322255 PMC7764238

[B55] von Wrede R. Bröhl T. Rings T. Pukropski J. Helmstaedter C. Lehnertz K. (2022a). Modifications of functional human brain networks by transcutaneous auricular vagus nerve stimulation: Impact of time of day. Brain Sci. 12, 546. 10.3390/brainsci12050546 35624933 PMC9139099

[B56] von Wrede R. Rings T. Bröhl T. Pukropski J. Schach S. Helmstaedter C. (2022b). Transcutaneous auricular vagus nerve stimulation differently modifies functional brain networks of subjects with different epilepsy types. Front. Hum. Neurosci. 16, 867563. 10.3389/fnhum.2022.867563 35814953 PMC9260140

[B57] von Wrede R. Rings T. Schach S. Helmstaedter C. Lehnertz K. (2021). Transcutaneous auricular vagus nerve stimulation induces stabilizing modifications in large-scale functional brain networks: Towards understanding the effects of taVNS in subjects with epilepsy. Sci. Rep. 11, 7906. 10.1038/s41598-021-87032-1 33846432 PMC8042037

[B58] von Wrede R. Surges R. (2021). Transcutaneous vagus nerve stimulation in the treatment of drug-resistant epilepsy. Aut. Neurosci. 235, 102840. 10.1016/j.autneu.2021.102840 34246121

[B59] Vonck K. Van Laere K. Dedeurwaerdere S. Caemaert J. De Reuck J. Boon P. (2001). The mechanism of action of vagus nerve stimulation for refractory epilepsy: The current status. J. Clin. Neurophysiol. 18, 394–401. 10.1097/00004691-200109000-00002 11709643

[B60] Wolf V. Kühnel A. Teckentrup V. Koenig J. Kroemer N. B. (2021). Does transcutaneous auricular vagus nerve stimulation affect vagally mediated heart rate variability? A living and interactive Bayesian meta-analysis. Psychophysiol 58, e13933. 10.1111/psyp.13933 34473846

[B61] World Health Organization (2019). Epilepsy. Geneva: World Health Organization.

[B62] Wu M.-L. Hu D.-M. Wang J.-J. Liu X.-L. Liu L. Li Y. (2021). Pre-and postoperative heart rate variability and vagus nerve stimulation in patients with drug-resistant epilepsy–A meta-analysis. Epilepsy Behav. 123, 108247. 10.1016/j.yebeh.2021.108247 34418640

[B63] Yap J. Y. Y. Keatch C. Lambert E. Woods W. Stoddart P. R. Kameneva T. (2020). Critical review of transcutaneous vagus nerve stimulation: Challenges for Translation to clinical practice. Front. Neurosci. 14, 284. 10.3389/fnins.2020.00284 32410932 PMC7199464

[B64] Yuan H. Silberstein S. D. (2016a). Vagus nerve and vagus nerve stimulation, a comprehensive review: Part I. Headache 56, 71–78. 10.1111/head.12647 26364692

[B65] Yuan H. Silberstein S. D. (2016b). Vagus nerve and vagus nerve stimulation, a comprehensive review: Part II. Headache 56, 259–266. 10.1111/head.12650 26381725

[B66] Yuen A. W. Sander J. W. (2017). Can natural ways to stimulate the vagus nerve improve seizure control? Epilepsy Behav. 67, 105–110. 10.1016/j.yebeh.2016.10.039 28152451

